# Neutrophil to lymphocyte ratio (NLR) as a prognostic marker for in-hospital mortality of patients with sepsis: A secondary analysis based on a single-center, retrospective, cohort study

**DOI:** 10.1097/MD.0000000000018029

**Published:** 2019-11-15

**Authors:** Jie Ni, Hongye Wang, Yue Li, Yimei Shu, Yihai Liu

**Affiliations:** a Department of Emergency, Nanjing Drum Tower Hospital, The Affiliated Hospital of Nanjing University Medical School.; b Nanjing Medical University, Nanjing, China.

**Keywords:** in-hospital mortality, neutrophil-to-lymphocyte ratio, sepsis

## Abstract

Neutrophil-to-lymphocyte ratio (NLR) has been reported to serve as a prognostic marker in inflammatory diseases. The purpose of this study was to evaluate the association of NLR at admission with in-hospital mortality in patients with sepsis presenting to emergency department.

This was a secondary analysis based on a single-center, retrospective, cohort study. Patients with sepsis admitted to an academic emergency department between January 2010 and January 2015 were enrolled. NLR of patients was analyzed from the hospital's electronic health record (EHR) system. A total of 174 adult patients, of which 80 (46.0%) died in hospital. The primary outcome was in-hospital mortality. Secondary outcome was 28-day mortality.

Contrary to previous studies, a larger NLR was found to have less odds of in-hospital mortality, as well as the presence of bacteremia. Patients who has severe/shock or had a history of chronic heart failure (CHF) had larger odds of death during hospital. Multivariate logistic regression analysis indicated that low NLR was an independent predictor of in-hospital mortality (OR = –0.98; 95% CI –0.96 to –0.99; *P* = .022). However, no correlation was found between the NLR and 28-day hospital mortality in patients with sepsis (*P* = .988). As a predictor of in-hospital survival, the area under curve (AUC) of the NLR was 0.622 (95%CI 0.54–0.71; *P* = .006) and the cut-off value was 9.11 with 0.551 sensitivity and 0.707 specificity.

NLR at admission was an independent predictor of in-hospital mortality of sepsis patients.

## 1 Introduction

Sepsis is a major public health issue and a leading cause of hospitalization and mortality in the developed world,^[1]^ with more than 1.5 million population diagnosed with sepsis in the USA and 250,000 Americans died from sepsis yearly. Even the survivors have long-term disabilities and readmission. Thus, it is significant to find the predictors of in-hospital CHF mortality of sepsis. A previous study found that patients with CHF had a higher in-hospital mortality (OR 2.45; 95%CI 1.22–4.88).^[2]^ Even after hospital discharge, septic patients with CHF had an increased 3-month and 1-year mortality. Another study reported that dementia acted as an independent mortality predictor in elderly sepsis patients.^[3]^ In addition, a prospective, observational, multicenter cohort study had identified other independent predictors of mortality in sepsis including age, APACHE II score, acute kidney injury (AKI) and thrombocytopenia.^[4]^

The neutrophil-to-lymphocyte ratio (NLR) is a readily available parameter that can be analyzed based on a complete blood count. NLR has previously been shown to predict adverse outcomes in oncology patients^[5]^ including lung,^[6]^ ovary,^[7]^ and breast^[8]^ malignancies. Despite a large number of evidences confirmed an association between NLR and mortality, the relationship between NLR and outcomes of sepsis patients was rarely investigated.

Three studies reported that elevated NLR was related to a poor long-term prognosis of sepsis patients and correlated with the severity of the disease.^[9–11]^ However, whether NLR increased in-hospital mortality remained controversial. Our study was to evaluate whether NLR was associated with in-hospital mortality in patients with sepsis.

## 2 Materials and methods

### 2.1 Study design

All participants in our study were enrolled from an emergency department of a large tertiary care center, which were publicly available in Dryad database. Briefly, all individuals presenting from January 2010 to January 2015 had their medical records, including comorbidities, vital signs, laboratory data and resuscitation parameters queried via the hospital's EHR system. The study was conducted in accordance with the Declaration of Helsinki and the informed consent was obtained from the participants or from their families if very sick to read the consent. Details regarding the aims and inclusion criteria of the trial have been described previously.^[2]^

### 2.2 Sepsis

According to the updated guide-Sepsis-3, sepsis is now defined as an acute increase in Sequential Organ Failure Assessment score of 2 or more from baseline in a patient with suspected infection.^[12]^ A documented or a presumed infection was defined as with 2 or more of the following:

(1) temperature >38°C or < 36°C,
(2) heartrate of > 90 bpm,
(3) respiratory rate of > 20 breaths/minute,
(4) white blood cell count > 12 × 109 /L.

Patients who were younger than 18 years old, pregnant, or presenting secondary to trauma were excluded.

The primary exposure of interest was NLR measured at admission. NLR was computed based on laboratory data as a ratio of neutrophil/lymphocyte values, which are recorded in the complete blood count, and patients were divided into survivors and nonsurvivors.

### 2.3 Data collection

The infection site was determined from the medical record, culture results (blood, sputum, urine, other fluids) and/or radiological data (such as chest X-rays). Vital signs were obtained from the Nursing record sheet while laboratory results from the hospital's EHR system. Information about medications used, time to their initiation and duration of their use was obtained by reviewing the scanned doctor's advice sheet.

The primary outcome was in-hospital mortality and the secondary outcome was 28-day mortality. 28-day mortality data were collected from the hospital medical records and telephone follow-up.

## 3 Statistical analysis

Statistical analyses were performed using SPSS V22.0 (IBM, USA). The distributions of the continuous and categorical indicators were described as mean ± SD and frequency/percentages, respectively. Pearson *χ*^2^ test was used to assess for statistical significance for the categorical variables, while the Student *t* test and the Mann-Whitney *U* test were used for the continuous indictors. A multivariable analysis was performed to examine the association between NLR and mortality in the septic population via a logistic regression. Receiver operating curve (ROC) was utilized to evaluate the predictive value of NLR as a predictor of in-hospital mortality of sepsis patients. All tests with *P* < .05 were interpreted as a significant difference.

## 4 Results

### 4.1 Population characteristics

A total of 174 patients with sepsis were included in the study, of which 80 (46.0%) died in hospital (Table 1). The mean age was 72.7 ± 13.9 and 72.9 ± 14.7 years old for survivors and non-survivors, respectively. There were more male patients among survivors than non-survivors (45.7% vs 32.5%). The survivor cohort had a higher percentage of hypertension, diabetes mellitus, dyslipidemia and chronic kidney disease (CKD), but a lower rate of coronary artery disease (CAD), chronic obstructive pulmonary disease (COPD) and CHF. However, only 1 common comorbidity, CHF, manifested a significant difference (39.4% vs 62.5%; *P* = .002). In respect to site of infection, no statistical differences were observed between 2 groups but urine infection and bacteremia. (*P* < .001; *P* = .001 respectively). Lung infections were more frequent and urine infections and bacteremia less frequent among non-survivors.

**Table 1:**
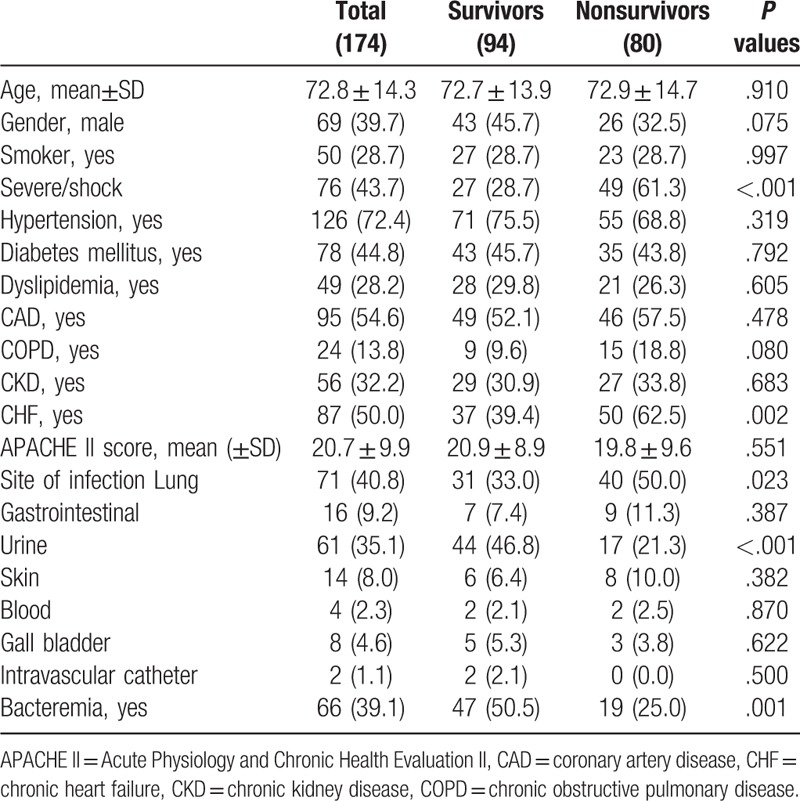
Demographics.

### 4.2 Laboratory tests and treatment

The 2 cohorts had comparable vital signs and laboratory values at presentation (Table 2). The non-survivor group had a lower heat rate and temperature, which may due to the difference in severity of sepsis. Interestingly, phosphate, magnesium, and potassium levels were higher in the non-survivors arm.

**Table 2:**
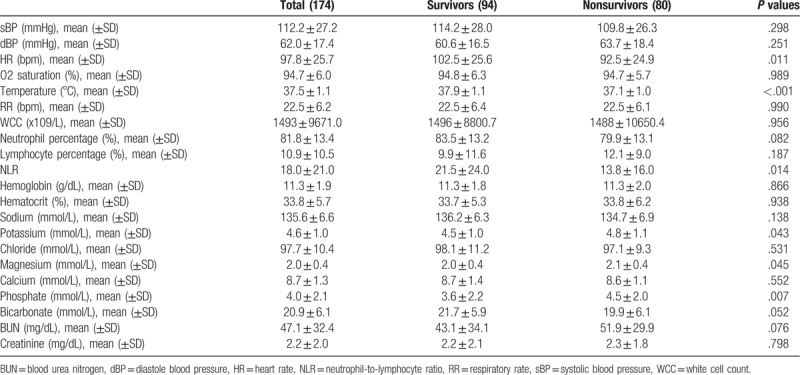
Vital signs and laboratory parameters.

As for treatment, non-survivors had been administrated more vasopressors, dobutamine and intubation as compared with survivors, indicating that non-survivors had experienced more active treatments (Table 3).

**Table 3:**
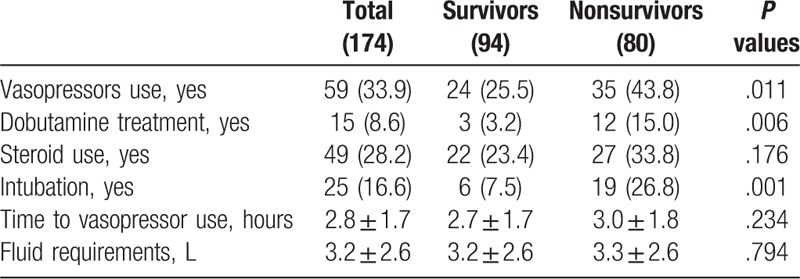
Sepsis treatment variables.

### 4.3 In-hospital mortality

**Figure 1:**
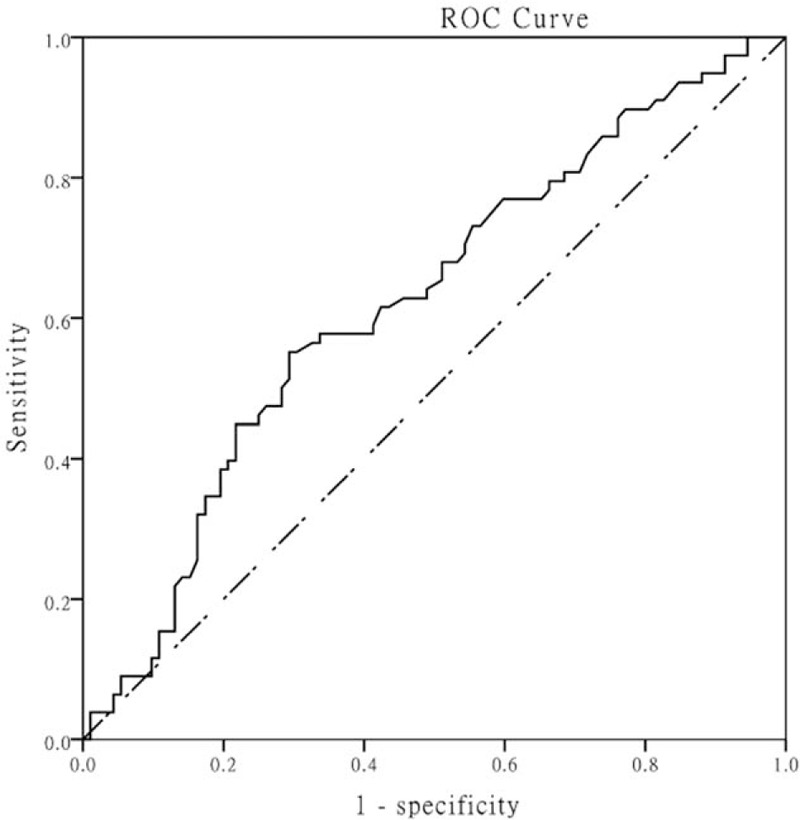
Receiver operating curve of neutrophil-to-lymphocyte ratio as a predictor. Area under curve was 0.62 (0.54–0.71) with cut-off of 9.11.

A multivariable logistic model was constructed to determine the association between NLR and in-hospital mortality adjusted for the clinically relevant and statistically significant variables. A higher NLR was found to have less odds of in-hospital mortality (OR = -0.98; 95%CI –0.96 to –0.99), as well as bacteremia (OR = 0.32; 95% CI 0.15–0.67). Meanwhile, it was shown that patients who were severe sepsis/shock (OR 4.99; 95% CI 2.39–10.39) or had a history of CHF (OR = 2.19; 95% CI 1.08–4.46) had larger odds of death. However, no statistically relevance was observed between NLR and 28-day mortality after adjusting for other confounders. The results of the multivariate analysis were shown in Table 4. To evaluate the value of the NLR as a predictor of in-hospital mortality of sepsis patients, a ROC curve was plotted (Fig. 1). The AUC of the NLR for hospital mortality was 0.62 (95%CI 0.54–0.71, *P* = .006) and the cut-off value was 9.11 with 0.551 sensitivity and 0.707 specificity.

**Table 4:**
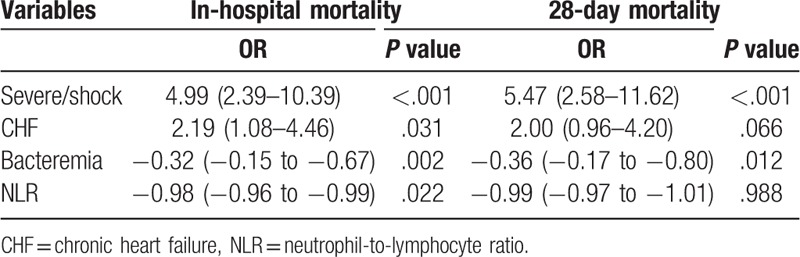
multivariate logistic regression analysis of NLR and in-hospital mortality.

## 5 Discussion

Sepsis was characterized by striking rate of mortality, which demanded efforts to further clarify the determinants and develop strategies in terms of quality of care and care transitions to prevent this outcome. We found that the non-survivors had a relatively lower NLR, with a 0.1-fold reduction in mortality for every 10-unit increase (OR = 0.99; 95% CI 0.97–1.01), and independent of CHF and disease severity. As a predictive factor, NLR has a statistically significant ROC (AUC = 0.62; 95%CI 0.53–0.71) with the cut-off value of 9.11.

A recent study investigating the relation between NLR and AKI has found that survivors has a higher NLR with significant statistical difference but whether NLR could be a predictor of in-hospital mortality of sepsis was not reported.^[13]^ Another study regarded NLR as a discrete variable, and reported that 1st quartile had the largest in-hospital mortality while 4th quartile the lowest mortality, suggesting an inverse relationship.^[10]^ What is more, septic shock patients who died before day 5 had a lower NLR at admission than survivors.^[14]^ Our results also suggested that NLR was negatively correlated with in-hospital mortality which could be used as an independent predictor. A report found that patients with sepsis had higher baseline NLR values, but there was no significant relationship between NLR and 28-day mortality.^[15]^ Nevertheless, our result confirmed that there was no relation between NLR and 28-day mortality. Even a study indicated that NLR was an independent predictor of 28-day mortality, it only included the severe sepsis or shock population.^[10]^

Consistent with previous reports,^[2]^ the patients with CHF had more than 2-fold odds of dying in the hospital than non-CHF patients. Cardiac dysfunction in patients with sepsis manifested as a reduced EF, which prevented the heart from increasing its cardiac output and meeting the metabolic demands needed to fight off the infection. Severe/shock was also associated with in-hospital mortality, which was easy to understand. The more severe the disease was, the more difficult it was to control the inflammation and the worse the prognosis would be. As for bacteremia, it may be a protective factor, which could be attributed to patients with bacteremia receiving more timely antibiotic treatments and fluid infusion.

Both widespread activation and dysfunction of the immune system are part of pathophysiological process of sepsis.^[16]^ NLR shows a balance between neutral and lymphoid, and it is a sign of systemic inflammation. On one hand, low circulating neutrophils increases hospital mortality. Sepsis patients with insufficient numbers of circulating neutrophils could have difficulties to start the innate immune response. Besides, lower NLR may indicate that increased neutrophil adhesion to the vascular endothelium, which induced endothelial damage.^[17]^ Patients with lower NLR often suffer from neutropenia, a risk factor for bacterial infection, and could not fight for subsequent severe sepsis and septic shock.^[18]^ On the other hand, lymphocytosis may initiate an excessive adaptive immune response where activation of massive T cells may aggravate tissue damages, followed by organ failure and death.^[19]^

However, our study had several limitations. First, this study was a single center, retrospective case analysis, and it was difficult to confirm causality. Second, although we adjusted the indicators that influenced outcomes in the multivariate logistic model, baseline differences in patient populations would influence the conclusions of this study. Thirdly, we only recorded the NLR at admission, and it may make more sense to monitor dynamical NLR changes. Last but not least, it was required to carefully interpret NLR because NLR was influenced by medications and morbidities that could affect the neutrophil and lymphocyte count, especially steroids use accounting for 28.2% of all patients. Even though the percentage of steroids use was not statistically different between 2 groups (*P* = .176), the influence should be carefully considered.

## 6 Conclusion

The NLR measured at admission of sepsis patients was an independent predictor of in-hospital mortality. The mechanism underlying the relationship has not yet to be fully elucidated and should be the focus of future prospective clinical research.

## Acknowledgments

The authors were very grateful to the data providers of the study.

## Author contributions

**Formal analysis:** Yihai Liu

**Investigation:** Jie Ni

**Methodology:** Yihai Liu

**Resources:** Jie Ni

**Software:** Hongye Wang

**Supervision:** Yue Li

**Validation:** Jie Ni

**Visualization:** Yimei Shu

**Writing-original draft:** Yihai Liu

**Writing-review & editing:** Jie Ni

**Conceptualization:** Jie Ni.

## References

[1] Prescott HC Osterholzer JJ Langa KM Late mortality after sepsis: propensity matched cohort study. BMJ 2016;353:i2375. 27189000 10.1136/bmj.i2375 PMC4869794

[2] Abou Dagher G Hajjar K Khoury C Outcomes of patients with systolic heart failure presenting with sepsis to the emergency department of a tertiary hospital: a retrospective chart review study from Lebanon. BMJ Open 2018;8:e022185. 10.1136/bmjopen-2018-022185 PMC6074621 30068620

[3] Bouza C Martinez-Ales G Lopez-Cuadrado T The impact of dementia on hospital outcomes for elderly patients with sepsis: a population-based study. PLoS One 2019;14:e0212196. 30779777 10.1371/journal.pone.0212196 PMC6380589

[4] Martin-Loeches I Guia MC Vallecoccia MS Risk factors for mortality in elderly and very elderly critically ill patients with sepsis: a prospective, observational, multicenter cohort study. Ann Intensive Care 2019;9:26. 30715638 10.1186/s13613-019-0495-x PMC6362175

[5] Zahorec R Ratio of neutrophil to lymphocyte counts--rapid and simple parameter of systemic inflammation and stress in critically ill. Bratisl Lek Listy 2001;102:5–14. 11723675

[6] Sarraf KM Belcher E Raevsky E Neutrophil/lymphocyte ratio and its association with survival after complete resection in non-small cell lung cancer. J Thorac Cardiovasc Surg 2009;137:425–8. 19185164 10.1016/j.jtcvs.2008.05.046

[7] Cho H Hur HW Kim SW Pre-treatment neutrophil to lymphocyte ratio is elevated in epithelial ovarian cancer and predicts survival after treatment. Cancer Immunol Immunother 2009;58:15–23. 18414853 10.1007/s00262-008-0516-3 PMC11029845

[8] Azab B Bhatt VR Phookan J Usefulness of the neutrophil-to-lymphocyte ratio in predicting short- and long-term mortality in breast cancer patients. Ann Surg Oncol 2012;19:217–24. 21638095 10.1245/s10434-011-1814-0

[9] Liu X Shen Y Wang H Prognostic significance of neutrophil-to-lymphocyte ratio in patients with sepsis: a prospective observational study. Mediators Inflamm 2016;2016:8191254. 27110067 10.1155/2016/8191254 PMC4823514

[10] Hwang SY Shin TG Jo IJ Neutrophil-to-lymphocyte ratio as a prognostic marker in critically-ill septic patients. Am J Emerg Med 2017;35:234–9. 27806894 10.1016/j.ajem.2016.10.055

[11] Liu Y Zheng J Zhang D Neutrophil-lymphocyte ratio and plasma lactate predict 28-day mortality in patients with sepsis. J Clin Lab Anal 2019;33:1–6. 10.1002/jcla.22942 PMC6757133 31265174

[12] Singer M Deutschman CS Seymour CW The third international consensus definitions for sepsis and septic shock (sepsis-3). JAMA 2016;315:801–10. 26903338 10.1001/jama.2016.0287 PMC4968574

[13] Bu X Zhang L Chen P Relation of neutrophil-to-lymphocyte ratio to acute kidney injury in patients with sepsis and septic shock: a retrospective study. Int Immunopharmacol 2019;70:372–7. 30852292 10.1016/j.intimp.2019.02.043

[14] Riche F Gayat E Barthelemy R Reversal of neutrophil-to-lymphocyte count ratio in early versus late death from septic shock. Crit Care 2015;19:439. 26671018 10.1186/s13054-015-1144-x PMC4699332

[15] Salciccioli JD Marshall DC Pimentel MA The association between the neutrophil-to-lymphocyte ratio and mortality in critical illness: an observational cohort study. Crit Care 2015;19:13. 25598149 10.1186/s13054-014-0731-6 PMC4344736

[16] Angus DC van der Poll T Severe sepsis and septic shock. N Engl J Med 2013;369:840–51. 23984731 10.1056/NEJMra1208623

[17] Bermejo-Martin JF Tamayo E Ruiz G Express, groups G. Circulating neutrophil counts and mortality in septic shock. Crit Care 2014;18:407. 24524810 10.1186/cc13728 PMC4057453

[18] Legrand M Max A Peigne V Survival in neutropenic patients with severe sepsis or septic shock. Crit Care Med 2012;40:43–9. 21926615 10.1097/CCM.0b013e31822b50c2

[19] Rimmele T Payen D Cantaluppi V Immune cell phenotype and function in sepsis. Shock 2016;45:282–91. 26529661 10.1097/SHK.0000000000000495 PMC4752878

